# The burden of pancreatic cancer is rising in Brazil

**DOI:** 10.1590/1516-3180.2019.13752231019

**Published:** 2020-01-13

**Authors:** Paulo Andrade Lotufo

**Affiliations:** I MD, DrPH. Full Professor, Department of Internal Medicine, Faculdade de Medicina da Universidade de São Paulo (FMUSP), São Paulo (SP), Brazil.

In a recent Editorial in this Journal, we reported that in Brazil among the middle-aged population stratum, the mortality rates due to pancreatic cancer are increasing at a pace of 2% per year for both sexes.^1^ For this trend to be better understood, we have analyzed the data relating to incidence, mortality and case-fatality rates for pancreatic cancer published up to 2019.

It is important to emphasize two points concerning the methods and conclusions relating to pancreatic cancer epidemiology. Firstly, there are inconsistencies in the information published worldwide. Secondly, despite the discrepancies between the methods, there is no doubt that the burden of pancreatic cancer is increasing in Brazil.

Geographical and temporal comparisons of incidence and mortality rates for pancreatic cancer are not easy to estimate. The reasons for this are that the symptoms of pancreatic cancer are only rarely identified in the early stage, and that the disease is usually at an advanced stage when diagnosed.^2^ Pancreatic cancer is the most fatal of all major cancers, with a median survival time of only approximately six months and five-year relative survival of only 4%.^3^

From 1980 to 2013, all-age pancreatic cancer mortality rates among men increased in Europe, Brazil, Japan and South Korea but declined in Australia, Canada, Mexico and the United States. On the other hand, among women, mortality increased in Europe, Brazil, the United States, Japan and South Korea but decreased in Canada and Mexico. The highest pancreatic cancer death rates for both sexes in 2012 were observed in eastern European countries and Japan.^4^

The age-standardized rates due to pancreatic cancer from 2000 and 2014 showed that the highest rates were recorded for the states of the central-western region, for both genders. The temporal trends of the age-adjusted rates were different for women, with a non-significant annual percentage change of 0.4% (95% confidence interval: -0.2 to 1.0) from 2000 to 2014, without any inflection of the curve. On the other hand, for men, a significant upward trend was observed from 2000 to 2004, with an annual percentage change of 3.7% (95% confidence interval: 0.6 to 7.0), followed by a period of stability, but increasing again from 2010 to 2014, with an annual percentage change of 2.7% (95% confidence interval: 0.2 to 5.2).^5^

A comparison of incident rates for pancreatic cancer among 43 countries revealed that in Brazil, there were non-statistically significant annual percentage increases in age-adjusted incident rates for pancreatic cancer, of 2.4% for men, and 0.1 % for women from 1988 to 2007 (the most recent data available). For men, the increase in the rate was constant over the period of observation and it occurred at the same pace for all birth cohorts analyzed.^6^ Another analysis on age-standardized incidence rates, using the GloboCan database, revealed that the highest incidence rate rises among men in Brazil were between 1998 and 2007, with an average annual percentage change of 10.4% (95% confidence interval: 0.8 to 21).^7^ The number of hospitalizations due to pancreatic cancer (not the incidence rate) within the Brazilian National Health System doubled from 2005 to 2012 for both sexes, from 2.4/100,000 to 4.5/100,000, with the highest increase in the states of the northeastern region.^8^

The importance of this demonstration of the increase in the burden of pancreatic cancer is that there are no detectable risk factors for this neoplasm.^9^ People with pancreatic cancer have not benefited from recent improvements in overall survival relating to genetic profiling (“precision medicine”),^10^ and no screening tool has been proven to be effective for early diagnosis of pancreatic cancer.^11^
